# Hemochromatosis Mimicked Gaucher Disease: Role of Hyperferritinemia in Evaluation of a Clinical Case

**DOI:** 10.3390/biology11060914

**Published:** 2022-06-15

**Authors:** Carmela Zizzo, Irene Ruggeri, Paolo Colomba, Christiano Argano, Daniele Francofonte, Marcomaria Zora, Emanuela Maria Marsana, Giovanni Duro, Salvatore Corrao

**Affiliations:** 1 Institute for Biomedical Research and Innovation (IRIB) National Research Council (CNR), Via Ugo la Malfa 153, 90146 Palermo, Italy; paolo.colomba@irib.cnr.it (P.C.); daniele.francofonte@irib.cnr.it (D.F.); marcomaria.zora@irib.cnr.it (M.Z.); emanuelamaria.marsana@irib.cnr.it (E.M.M.); giovanni.duro@irib.cnr.it (G.D.); 2 Department of Internal Medicine, National Relevance and High Specialization Hospital Trust ARNAS Civico, Di Cristina, Benfratelli, 90127 Palermo, Italy; ireneruggeri@icloud.com (I.R.); christiano.argano@unipa.it (C.A.); salvatore.corrao@unipa.it (S.C.); 3 Dipartimento di Promozione della Salute, Materno Infantile, Medicina Interna e Specialistica di Eccellenza “G.D’Alessandro”, PROMISE, University of Palermo, 90127 Palermo, Italy

**Keywords:** Gaucher disease, hyperferritinemia, hemochromatosis, misdiagnosis

## Abstract

**Simple Summary:**

In this paper, we describe the problem of diagnostic delay in Gaucher disease through the evaluation of a clinical case and how some clinical–instrumental parameters, such as hyperferritinemia should trigger an alarm bell in clinicians, inducing the clinical suspicion of Gaucher disease. In the described case of a 38-year-old man, the first diagnosis of hemochromatosis was updated to type 1 Gaucher disease years later. Analysis of the clinical case showed that the first diagnosis of hemochromatosis had been made almost exclusively based on a finding of high levels of ferritin and splenomegaly. Type 1 Gaucher disease and hereditary hemochromatosis show common clinical features, such as asthenia, joint pain, splenomegaly and hyperferritinemia. For this reason, in the presence of “unexplained hyperferritinemia” it is necessary to consider Gaucher disease in differential diagnosis, even if few typical signs and symptoms of the disease are present. The combination of a careful clinical history with simple first-level investigations may be sufficient to make differential diagnosis, also including rare conditions, such as Gaucher disease. Misdiagnosis and delayed diagnosis in metabolic diseases can lead to irreversible organ damage and a delayed start of specific therapy for these patients.

**Abstract:**

Gaucher disease is a disorder of lysosomes caused by a functional defect of the glucocerebrosidase enzyme. The disease is mainly due to mutations in the GBA1 gene, which determines the gradual storage of glucosylceramide substrate in the patient’s macrophages. In this paper, we describe the case of a 38-year-old man who clinically presented with hyperferritinemia, thrombocytopenia, leukopenia, anemia and mild splenomegaly; a diagnosis of hemochromatosis was made 10 years earlier. Re-evaluation of the clinical case led to a suspicion of Gaucher disease, which was confirmed by enzymatic analysis, which was found to be below the normal range, and genetic evaluation, which identified compound heterozygosity N370S/RecNciI. We know that patients suffering from Gaucher disease can also have high ferritin levels. Even if the mechanism underlying the changes in iron metabolism is not yet elucidated, the chronic mild inflammatory state present in these patients probably causes the storage of ferritin in macrophages, resulting in hyperferritinemia. Therefore, in the presence of few typical signs and symptoms of the disease should raise an alarm bell in the clinicians, inducing clinical suspicion of Gaucher disease. Misdiagnosis and diagnostic delay in metabolic diseases could cause irreversible organ damage and delay the start of specific therapy for these patients.

## 1. Introduction

Gaucher disease (GD, OMIM #230800, ORPHA355) is a rare, autosomal, recessive genetic disease caused by mutations in the GBA1 gene located on chromosome 1 (1q21) coding for the lysosomal enzyme glucocerebrosidase (GCase, also called glucosylceramidase or acid β-glucosidase 1, EC: 4.2.1.25) [1,2,3].

Gaucher disease is the most common sphingolipidosis, with an incidence of around 1/40,000 to 1/60,000 births in the general population, although its incidence can reach 1/800 births in the Ashkenazi Jewish population [4,5]. GD is one of the 60 lysosomal disorders described to date, and it is caused by a deficiency of the lysosomal enzyme glucocerebrosidase, which hydrolyzes glucosylceramide (GlcCer) into ceramide and glucose. This impairment leads to an accumulation of its substrate, glucosylceramide, in macrophages, inducing their transformation into Gaucher cells that infiltrate bone marrow, the spleen, the liver and other organs. Gaucher cells are considered the main protagonists with respect to disease symptoms [1]. The phenotype is variable, but three clinical forms have been identified: type 1 is the most common and typically causes no neurological damage, whereas types 2 and 3 are characterized by neurological impairment. However, these distinctions are not absolute, and it is increasingly recognized that neuropathic GD represents a phenotypic continuum, ranging from extrapyramidal syndrome in type 1 at the mild end to hydrops fetalis at the severe end of type 2 [6]. Due to the rarity and heterogeneity of the clinical picture resulting in varying disease severity, GD can be misdiagnosed with other more common pathologies. Hemochromatosis is a disease caused by defects in the hereditary mechanisms of iron metabolism regulation, leading to its progressive accumulation and to the development of serious organ damage in its most advanced stage. It can be underestimated and sometimes also overestimated, similar to many types of liver diseases. However, different conditions affecting other organs can reveal high iron levels that simulate hemochromatosis. Among these, Gaucher type I disease can also manifest as hyperferritinemia [7], leading to a misdiagnosis of hemochromatosis. Total clinical features of hyperferritinemia or its association with HFE gene mutations are unknown. In this sense, it must be considered whether hyperferritinemia can be used as a biomarker in GD.

In this paper, we present the case of a 38-year-old man with hyperferritinemia, thrombocytopenia, leukopenia, anemia, mild splenomegaly and no other signs or symptoms typical of GD. Splenomegaly, along with other clinical signs, such as asthenia, oral aphthosis and diffuse folliculitis of the trunk, had already occurred 10 years before and led to an initial suspicion of hemochromatosis. Re-evaluation of the clinical case led to a clinical hypothesis of GD, which was confirmed by enzymatic analysis, which was found to be below normal range, and by genetic analysis that identified compound heterozygous N370S/RecNciI. Furthermore, in this subject, chitotriosidase was also found to be above the normal range, supporting the diagnosis of GD.

## 2. Materials and Methods

### 2.1. Sample Collection

Peripheral blood of the patient was collected using EDTA as an anticoagulant and dried in specific absorbent paper spot (dried blood spot (DBS)). The genetic and enzymatic studies performed at the Center for Research and Diagnosis of Lysosomal Storage Disorders of CNR in Palermo were approved by the Hospital Ethics Committee of the University of Palermo. Signed informed consent was obtained from the patient.

### 2.2. Glucocerebrosidase Activity Assay

Acid β-glucosidase analysis was determined by the dried blood filter paper test (DBFP) described by Chamoles et al. [8], with modifications (unpublished data).

### 2.3. Chitotriosidase Assay

According to the literature [9], chitotriosidase activity analysis was performed on dried blood spots.

### 2.4. DNA Extraction

DNA extraction was performed using a Qiagen EZ1 advanced XL automatic extractor, and an EZ1 Advanced XL DNA investigator card (a programmed card containing protocols for DNA extraction by filter paper) was used in combination with the EZ1 DNA investigator kit. Quantification of genomic has required the use of an Eppendorf D30 biophotometer.

### 2.5. PCR and Sequencing

Because of the non-functional pseudogene GBAP, which has a high homology of sequence with the functional GBA1 gene, we used a long PCR approach projected after extensive study of the literature to identify specific mutations of GBA1 and GBAP recombinant alleles. The approach consists of carrying out the amplification of the gene, using two pairs of primers that amplify two macroregions from exon 1 to intron 5 and from intron 5 to exon 11 by Biotech-Rabbit high-fidelity long-range DNA Polymerase. Sequencing primers were projected to sequence the exons of GBA1 and large flanking intronic regions. PCR products were purified and sequenced by Eurofins Genomics.

### 2.6. Multiplex Ligation Probe Amplification

A search for large deletions/insertions in the GBA1 gene was performed by multiplex ligation probe amplification (MLPA) with a SALSA MLPA Probemix P338-B1 GBA kit. The kit contains 24 MLPA probes with amplification products between 155 and 382 nucleotides (nt), including nine probes for the GBA1 gene and three flanking probes. Probes for exons 2, 5 and 11 were missing.

### 2.7. Glucosylsphingosine Determination

Detection of glucosylsphingosine (Lyso Gb1) was performed on dried blood spots by LC-MS/MS technique [10,11,12,13]. Lyso-Gb1 was quantified by Centogene.

## 3. Results

**Figure 1:**
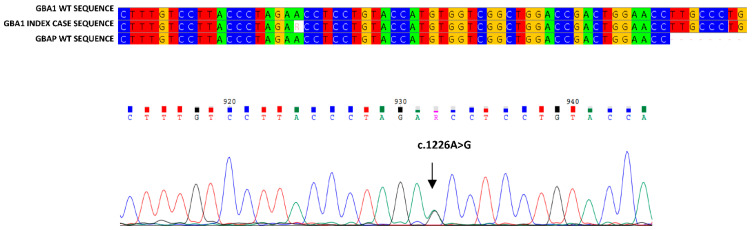
**Alignment of the index case sequence with GBA1 and GBAP wt reference sequences**. The figure shows the part of sequence in which the N370S mutation was found in this patient, that aligns with the reference sequences; its electropherogram is also present.

**Figure 2:**
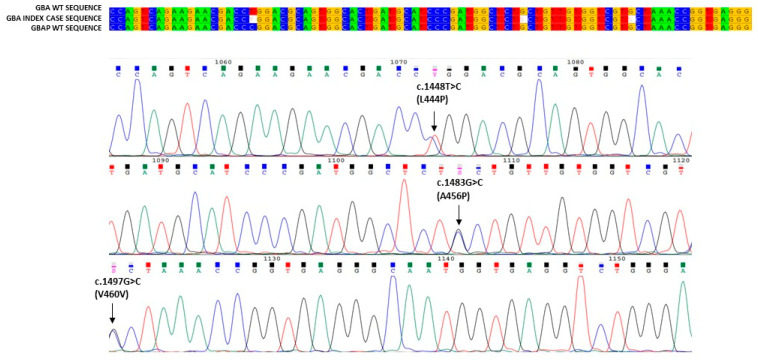
**Alignment of the index case sequence with GBA1 and GBAP wt reference sequences.** The figure shows the part of the patient’s sequence in which RecNci I (L444P + A456P + V460V) recombination falls, in alignment with the reference sequences; its electropherogram is also present.

In this paper, we describe the case of a 38-year-old man presenting with the coexistence of a clinical triad: hyperferritinemia, thrombocytopenia and splenomegaly. The patient also reported asthenia, oral aphthosis and diffuse folliculitis of the trunk, which had been presenting for several years. Due to these clinical symptoms, he had been subjected to periodic laboratory and instrumental investigations for about 10 years. Autoimmune diseases at national reference centers had been excluded, and hemochromatosis had been supposed on clinical basis. This clinical suspicion was ruled out at a highly specialized Italian reference center for hemochromatosis, where all instrumental and genetic tests were carried out. However, the clinical triad persisted without explanation. For this reason, a diagnostic process was started. We identified mild thrombocytopenia (139,000/µL platelets (the lowest value had been 90,000 in previous years)), significant lymphocytopenia (810/µL circulating lymphocytes), mild reduction in cholinesterase (4338 U/L; the normal value: 5320–12,920 U/L), elevated plasma ferritin (1570 µg/L; normal value: 24–336 µg/L), serum transferrin saturation (48%; normal value: 20–50%) and increased serum Beta2 macroglobulin levels (2.67 mg/L; normal value: 1.09–2.53 mg/L). Immunologic tests to identify viruses resulted in no relevant findings. Flow cytometry analysis confirmed marked lymphocytopenia of all T, B and NK lymphocytes. Nuclear magnetic resonance (RMN) revealed a splenomegaly (longitudinal diameter 17 cm, sagittal diameter 15 cm), a normal volume and signal intensity of the liver, and signal variations of dorsal–lumbar vertebrae of the sacrum and iliac bone characterized by a signal reduction with a variegated appearance at the level of the iliac bone, such as yellow medullar variation, normal lymph nodes and a normal signal of intra-abdominal organs, were identified. The results of these analyses suggested a diagnosis of GD. In order to confirm this clinical suspicion, we performed an enzymatic analysis of glucocerebrosidase, which was below the normal range (0.6 nmol/h/mL; normal range: ≥2.5 nmol/h/mL), as well as a chitotriosidase assay, which revealed high activity (606.12 nmol/h/mL; normal range: 0.0–50.0 nmol/h/mL). In order to validate these data evocating GD, we carried out genetic analysis. Our study of the GBA1 gene identified a c.1226A > G mutation (Figure 1) and the allelic recombination RecNci I (Figure 2) [14,15,16,17]. The former is an adenine-to-guanine transition at the 1226 nucleotide of cDNA, leading to an amino acid substitution (p.N409S) [18] also known as N370S-removing signal peptide; the latter is an allelic recombination event with the GBAP c. [1448T > C; 1483 G > C; 1497 G > C] [19] also known as p. [L483P, A495P, V499V] or (L444P + A456P + V460V)-removing signal peptide. In order to exclude large insertions or deletions, we also performed multiplex ligation-dependent probe amplification (MLPA) in the patient GBA1 gene, which afforded a negative result. Analysis of Lyso Gb1, considered a storage product in Gaucher disease, was also found to be pathological, further confirming the initial diagnostic suspicion. Nevertheless, in order to corroborate the compound heterozygous N370S/RecNciI, we carried out a family study on DNA of the proband parents, which identified N370S in the GBA1 gene of the proband’s mother and RecNciI: (L444P + A456P + V460V) in the GBA1 gene of the proband’s father. Both parents presented with normal acid β-glucosidase activity (Table 1). In this patient, enzymatic analysis revealed levels the normal range in terms of symptomatology related to the disease, high levels of Lyso Gb1 and compound heterozygosity proven by familial study, suggesting that this individual is affected by GD.

## 4. Discussion

Although GD is considered one of the most common lysosomal storage disorders, it remains an extremely rare disease. The gradual onset of clinical phenotypes and the high clinical heterogeneity could explain diagnostic delays and misdiagnosis in patients affected by GD. Several factors are probably involved in the variability of age at onset and in the severity of symptoms. These include the dysregulation of the autoimmune system through the overexpression of some proinflammatory cytokines, such as IL8, IL6, IL1 alpha, TNFalpha, M-CSF, the monocyte-macrophage activation marker sCD14 and the macrophage inflammation proteins MIP-1alpha and MIP-1beta [20,21,22]. They have been found to be potential causes of clinical severity and disease expression. Hyperferritinemia without accumulation of iron in the organs, which is an expression of an autoimmune system alteration, can also be observed in subjects suffering from GD [23]. Its involvement, as well as its role as a biomarker in GD, is unclear and not yet defined. Hyperferritinemia is thought to be a consequence of the storage of iron in Gaucher cells; recent studies suggest that macrophages are directly involved in serum ferritin production [24], and some newly diagnosed patients with GD present with massive infiltration of iron-stained Gaucher cells in the bone marrow [25]. Other studies showed that hyperferritinemia is an expression of high serum levels of hepcidin. Hyperferritinemia inhibits macrophage iron recovery, causing iron accumulation. Iron accumulation is influenced by inflammatory cytokines [26].

In this paper, we described the case of a 38-year-old man who received a first diagnosis of hemochromatosis, which was updated to type 1 GD years later. Analysis of clinical case showed that the first diagnosis was made almost exclusively based on a finding of high levels of ferritin and splenomegaly [27]. Type 1 GD and hereditary hemochromatosis present with common clinical features, such as asthenia, joint pain, splenomegaly and hyperferritinemia [28]. For this reason, in the presence of hyperferritinemia, it is necessary to consider GD in differential diagnosis. Usually, the presence of hyperferritinemia with a transferrin saturation rate >60% suggests hereditary hemochromatosis, whereas a value of 45–60% suggests other possible diseases consistent with iron overload. Instead, when the percentage of transferrin saturation is normal, hyperferritinemia can be associated with hepatic cytolysis, hemolysis, cancer, hyperthyroidism or chronic alcohol abuse, whereas a low transferrin value suggests the presence of an inflammatory syndrome [23]. Although the precise mechanism underlying changes in iron metabolism in GD has not yet been elucidated, high ferritin levels can also be found in GD. Therefore, in the presence of “unexplained hyperferritinemia”, even if few typical signs and symptoms of the disease are present, the combination of a careful clinical history with simple first-level investigations may be sufficient to make a differential diagnosis, also including rare conditions, such as GD.

When GD is suspected, further investigations should be adjusted based on the strength of the clinical suspicion. Analysis of enzymatic activity should be performed as a first-line screening strategy [29,30], followed by genetic analysis and lyso-Gb1 detection if enzymatic analysis results are positive [7]. Misdiagnosis and delayed diagnosis in metabolic diseases can lead to irreversible organ damage and delayed start of specific therapy for these patients.

## 5. Conclusions

The clinical diagnosis of GD, similar to those of other lysosomal storage disorders, is notoriously difficult because clinical signs and symptoms of this disease overlap with those of other common conditions. GD is often diagnosed at a later life stage and only after consultation with several specialists with experience with respect to the syndromic phenotypes of the same disease. Thus, the variability of the clinical phenotypes of GD and the overlap with other conditions represent an objective barrier to the timely identification of affected individuals, which may lead to an underestimation of the true prevalence of this condition. Our observations confirm the inherent difficulty in diagnosis of Gaucher disease on clinical grounds and suggest the potential of enzymatic and genetic tests in patients with high levels of hyperferritinemia.

## Figures and Tables

**Table 1:** **Clinical and molecular data of index case and his parents**. Values below the normal range and the name of GBA1 mutations are indicated in bold. Normal values of glucocerebrosidase activity assayed in whole blood are ≥2.5 nmol/h/mL.

Kinship	Age	Gender	GCase Activity	GBA1 Mutations		Status
Index case	38	M	0.6	N370S	RecNci I	Hyperferritinemia, thrombocytopenia, splenomegaly
Mother	66	F	5.6	N370S	wt	Asymptomatic
Father	67	M	4.8	wt	RecNci I	Asymptomatic

## Data Availability

Not applicable.
